# Addressing Clinical Ambiguity in Breast Density Assessment: A Hybrid Multi-View Deep Learning Framework for BI-RADS B vs. C Classification

**DOI:** 10.3390/diagnostics16132044

**Published:** 2026-06-30

**Authors:** Bochra Triqui, Hicham Kaid-Slimane

**Affiliations:** 1 Computer Science and New Technologies Laboratory (CSTL), University Abdel Hamid Ibn Badis, Mostaganem 27000, Algeria; 2 Medical Imaging Center ‘ERAHMA’, Mostaganem 27000, Algeria; kaidslimaneh@gmail.com

**Keywords:** breast density classification, mammographic image analysis, multi-view deep learning, hybrid EfficientNet–UNet, explainable AI, clinical decision support, computer-aided diagnosis, Grad-CAM

## Abstract

**Background/Objectives:** Mammographic breast density assessment represents a crucial step in the detection of breast cancer, risk stratification, and lesion visibility. However, it is generally quite difficult to distinguish between intermediate density categories, especially BI-RADS B and C, because of the high inter-observer variability between radiologists. This hindrance encourages researchers to develop novel robust and interpretable automated techniques. Thus, a hybrid multi-view deep learning framework based on EfficientNet-B4 and U-Net for BI-RADS B vs. C classification is presented in this work. **Methods:** The proposed model utilizes the fusion of craniocaudal (CC) and mediolateral oblique (MLO) views at the feature level to capture complementary fibroglandular anatomical and tissue characteristics. Furthermore, the interpretability of the model is ensured with Grad-CAM, which highlights the regions relevant to decision-making. The proposed approach is evaluated on the RSNA mammography dataset, which consists of 23,513 images of 4796 patients, after a patient-wise split, for the purpose of preventing data leakage and guaranteeing a clinically realistic assessment. This protocol offers a more reliable evaluation than the image-by-image assessment strategies usually employed in previous studies. **Results:** The experimental outcomes obtained indicate an accuracy of 87% and an area under the curve (AUC) of 94.40%. These performance levels are consistent with those reported in recent research, suggesting competitive performance in this complex and difficult classification task. These enhancements are statistically significant compared to the assessed reference values, as confirmed using statistical analysis based on McNemar’s and DeLong’s tests. Furthermore, the qualitative evaluation carried out by experienced and certified radiologists corroborates the clinical pertinence of the highlighted regions. **Conclusions:** Furthermore, it is found that combining multi-view deep learning and explainable AI within the proposed framework is consistent with observed inter-observer variability and may support more consistent breast density assessment and clinical decision-making. However, further prospective multicenter validation is necessary before any clinical application.

## 1. Introduction

Breast cancer is the most frequently diagnosed type of cancer in women worldwide. Research has shown that, in 2022 alone, approximately 2.3 million new cases of this disease were diagnosed, along with 670,000 deaths, with a projected incidence of over 3.2 million cases by 2050 [1,2]. Critically, early breast cancer detection via screening mammography is an essential way of reducing mortality, with a five-year relative survival rate approaching 99% when the disease is discovered at a localized stage [3,4].

Importantly, breast density, defined as the relative ratio of fibroglandular tissue to adipose tissue visible via mammography, is an independent risk factor for breast cancer and can reduce the sensitivity of conventional mammography.

Several studies have revealed that combining tomosynthesis with digital mammography improves cancer detection in women with dense breasts [5,6,7]. It should also be noted that dense breasts increase the risk of tumors and can even mask abnormalities, thus decreasing the sensitivity of mammograms, particularly for the intermediate categories B and C, which exhibit visual ambiguity and inter-reader variability [8,9,10]. Moreover, this variability complicates radiologists’ daily work, indicating a need for reliable automated systems that can be integrated into clinical workflows to provide radiologists with real-time decision support.

Moreover, according to the Breast Imaging Reporting and Data System (BI-RADS), developed by the American College of Radiology (ACR), breast composition can be classified into four distinct categories, namely, a (almost entirely fatty), B (scattered areas of fibroglandular density), C (heterogeneous and dense), and D (extremely dense), expressing growing breast density and declining mammographic sensitivity [11]. Critically, despite this standardization, visual assessment remains subject to moderate inter-observer variability, with kappa coefficients between 0.44 and 0.54, which can lead to discrepancies in risk stratification and clinical planning [12,13,14].

Breast density assessment is intrinsically dependent on inter-observer variability, particularly regarding the BI-RADS B vs. C transition. This represents the most clinically complex classification because of numerous subtle differences in fibroglandular tissue distribution. Conflicting interpretations between radiologists may then arise, which directly affects risk stratification, screening strategies, and follow-up recommendations. This continuous inter-observer variability indicates that it is necessary to develop automated systems capable of enhancing the reproducibility of the BI-RADS B vs. C classification. The choice of the BI-RADS B vs. C boundary is primarily justified by its well-documented high inter-observer variability, in which even expert radiologists often disagree. This makes it an ideal framework for evaluating clinically relevant decision support systems.

It is worth noting that early computer-assisted detection (CAD) methods, which relied on extracting textural or statistical features from an image, had rather limited performance due to parenchymal heterogeneity and inter-patient or protocol differences [15,16,17,18]. This has contributed to developing automated, data-driven diagnostic processing chains for the analysis of medical images. In recent years, the evolution toward deep learning models has enabled the design of robust, modular, and automated pipelines, incorporating explainability techniques such as Grad-CAM [13,19,20,21], which can be directly integrated into CAD systems or eHealth platforms, thus providing operational support for clinical decision-making.

It is widely acknowledged that breast density classification is essential for the assessment of breast cancer risk. In this context, numerous recent studies have investigated hybrid CNN and CNN-Transformer architectures with a view toward using them in medical image segmentation and breast lesion detection. For instance, the authors of [22] proposed the SwinUNet neural network architecture, which is a Swin Transformer–CNN hybrid with adaptive feature fusion designed for the segmentation of ultrasound breast lesions. In [23], the authors provided a comprehensive survey of architectural advances in deep CNNs, emphasizing all emerging challenges and applications. In [24], the authors introduced the Residual-SwinCA-Net framework, in which they integrated residual CNNs with Swin Transformers for malignant lesion segmentation in BUSI. Likewise, the authors of [25] developed and presented a channel-boosted residual CNN–Transformer with regional boundary learning for breast cancer detection. Finally, the authors of [26] evaluated the use of vision Transformers for the segmentation of medical images, highlighting the most recent trends and limitations.

Although these studies reported quite favorable performance in lesion segmentation or detection, they primarily concentrated on single-view or ultrasound data and did not explicitly address multi-view CC+MLO mammography for BI-RADS density classification with clinically validated interpretability. The method proposed in this work is designed to address this issue. The TwoViewDensityNet method [27,28] involves using either single-view models or basic averaging for multi-view fusion, restricting the capacity to capture complex inter-view correlations. In addition, many recent studies have shown that conventional deep learning models for mammographic classification generally depend on single-view analysis or simple fusion strategies, which may not fully exploit inter-view correlations in multi-view mammography (e.g., the Multi-View Swin Transformer (MV-Swin-T) highlights the limitations of simple multi-view fusion, while the multi-level CNN approach for BI-RADS categorization emphasizes the need for more effective view integration).

It is noteworthy that the performance reported in various studies is affected by the heterogeneity of evaluation protocols and dataset characteristics. More particularly, the image-by-image evaluation on selected datasets (for instance, INbreast, MIAS) cannot be directly compared to the patient-by-patient evaluation on large real-world cohorts such asRSNA. This generally provides a more realistic estimation of generalization performance, thus emphasizing the importance of consistent assessment protocols among studies.

The hybrid framework proposed in our work integrates the EfficientNet and U-Net architectures with the concatenation of multi-view features to capture local and global tissue patterns in mammographic images. The adopted method replicates the multi-view evaluation strategy used by radiologists by explicitly modeling the correlations between CC and MLO views, while surmounting the limitations of single-view or averaging-based merging methods.

In this context, we propose an automated, multi-view, deep learning-oriented pipeline, optimized for integration into a clinical CAD system, for BI-RADS B vs. C binary classification. This pipeline, which utilizes a multi-scale representation of mammographic images and multi-view fusion (CC and MLO), was applied to the RSNA Breast Cancer Screening Mammography Dataset. This design is in line with recent advances in deep convolutional neural networks for mammography, as reviewed by Abdelhafiz et al. [29], and is primarily intended to reduce inter-reader variability, improve the efficiency of radiological workflows, and provide interpretable outputs that can be directly exploited by radiologists. We focused on categories B and C due to their high visual ambiguity and inter-reader variability.

The reference standard considered in this study was established based on an agreement between several readers among expert radiologists, with the aim of achieving a more stable and clinically reliable field truth, especially for equivocal BI-RADS B vs. C cases.

This approach further highlights the clinical complexity of the BI-RADS B vs. C boundary. It is characterized by document-supported inter-observer variability.

The main contribution of this work lies in the clinical adaptation, multi-view learning design, and systematic patient-by-patient evaluation of a deep learning framework applied to the difficult problem of BI-RADS B vs. C distinction, using a large dataset. The originality of this study stems principally from the formulation of its clinically practice-oriented problem and its stringent evaluation scheme, rather than from any isolated architectural innovation.

The most significant contributions of this work are as follows:

1. We implemented rigorous signal preprocessing and normalization procedures, including selecting standard views, excluding implants, and balancing classes [21].
2. Patient-wise stratification was employed to prevent information leakage and ensure robust evaluation of independent signals.
3. We developed a multi-scale hybrid architecture, i.e., EfficientNet-B4 + U-Net, which is optimized for integration into a CAD pipeline and capable of capturing local and global signal characteristics while performing coherent multi-view fusion.
4. We integrated signal interpretability tools, such as Grad-CAM, to visualize regions contributing to the decision, thus building further confidence in potential clinical use [30,31].

The slightly smaller numerical performance noticed during the patient-wise evaluation should therefore not be viewed as a decreased potential of the model. Rather, it should be regarded as an expected result of a more realistic and clinically rigorous evaluation protocol.

The remainder of this article is structured as follows: Section 2 presents related work oriented toward system applications and representation learning, while Section 3 describes the materials, methods, and multi-view fusion protocols used in the CAD pipeline. Section 4 reports the experimental results and compares them to previously reported results. Finally, Section 5 discusses the clinical implications and some future perspectives, and Section 6 sets out the conclusions.

## 2. Related Work

Convolutional neural networks (CNNs) for automated breast density classification have been investigated in numerous recent studies [32,33,34]. Critically, early CAD methods relied primarily on manually designed features. They were limited by inter-patient variability, low image quality, and differences in acquisition protocols [35,36,37]. In addition, these methods were often unsuccessful in capturing complex tissue models or integrating seamlessly into clinical workflows.

Recent deep learning approaches have enhanced the interpretability of feature representations. In this regard, Lundervold [38] highlighted the challenges of generalization to heterogeneous datasets. Furthermore, to efficiently address the lack of labeled data, transfer learning from pre-trained convolutional neural networks has been widely applied [39]. Numerous interpretable multimodal approaches have therefore been developed [40]. However, to the best of our knowledge, few studies have expressly addressed the fusion of multi-view mammograms or provided clinically validated interpretability for BI-RADS B vs. C classification.

Notably, many CNN and CNN–Transformer methods have presented high lesion detection or segmentation performance. Nevertheless, their applicability to multi-view mammography for density classification remains limited.

Naz et al. [22] developed SwinUNet, a hybrid Swin Transformer–CNN optimized for breast ultrasound lesion segmentation. However, they did not address (CC+MLO) mammography or density classification.

Khan & Iqbal [23] conducted a CNN study providing an overview of architectural advances and emerging applications. This is theoretical work, and no multi-view fusion strategy was presented.

Naz and Khan [24] developed Residual-SwinCA-Net, a network integrating residual CNNs with Swin Transformers for segmenting malignant lesions in bioelectric magnetic resonance imaging (BMR). However, it does not handle inter-view correlations or B vs. C classification.

Mehmood et al. (2025) [25] developed a channel-boosted residual CNN transformer. It is particularly effective for lesion detection with regional boundary learning. However, it lacks explicit fusion (CC+MLO).

Khan et al. [26] conducted a review of vision transformers, highlighting the capabilities and limitations of Transformers regarding medical image segmentation. The authors report that no B vs. C classification has been clinically validated.

In the TwoViewDensityNet method developed by [27] and Nguyen et al. [28], simple averaging or single-view inputs are used for multi-view fusion. However, this approach is limited in its ability to capture inter-view correlations, particularly at the B vs. C boundary.

Proposed Method: Our EfficientNet–B4 + U-Net hybrid framework addresses these deficiencies by combining multi-scale feature extraction and explicit CC+MLO feature-level concatenation. Grad-CAM interpretability particularly scrutinizes relevant tissue regions, while patient-based assessment prevents information leakage. This procedure is particularly suited to the diagnostically challenging task of BI-RADS B vs. C classification. It offers a robust and clinically meaningful solution that is especially suitable for integration into CAD workflows.
**Method/Work****Multi-View Fusion****Architecture****Limitations/Comments**TwoViewDensityNet [27]AverageCNNIt does not capture inter-view correlations and has limited capacity for B vs. C differentiation.Nguyen et al., 2022 [28]Single-view/averageCNNIt has limited capacity for B vs. C differentiation and does not exploit complex multi-view fusion.Naz et al., 2026 (SwinUNet) [22]N/A (ultrasound only)Swin Transformer+ CNNIt is optimized for ultrasound and does not handle CC+MLO or B vs. C classification.Khan & Iqbal, 2025 [23]N/ACNN architectures (Survey)This work is theoretical. No multi-view fusion for BI-RADS was presented.Naz & Khan, 2025 (Residual-SwinCA-Net) [24]N/AResidual CNN + Swin TransformerThe focus was on BUSI lesion detection, not B vs. C or inter-view correlations.Mehmood et al., 2025 [25]N/AChannel-boosted residual CNN– TransformerIt is effective for lesion detection but lacks CC+MLO fusion capacity.Khan et al., 2025 [26]N/AVision Transformers (Review)No clinically validated B vs. C classification methods were presented.Proposed Method Feature concatenationEfficientNet-B4 + U-NetIt captures both local and global tissue, supports coherent CC+MLO fusion, and provides Grad-CAM interpretability and patient-wise evaluation.

Summary: Earlier approaches essentially focused on single-view analysis, ultrasound data, or theoretical insights. In contrast, our method explicitly addresses multi-view CC+MLO fusion for clinically ambiguous B vs. C borderlines. It combines interpretability and individualized assessment, offering robust and practical clinical relevance.

## 3. Materials and Methods

### 3.1. Software and Hardware Environment

The system was developed in Python 3.8 to ensure compatibility with scientific libraries. Modeling and training were performed using PyTorch version 2.9.0+cu126, a flexible framework offering GPU-optimized tensor operations and a high-level API for building neural networks. All steps were designed to facilitate future integration into a clinical CAD system, with an automated mammography signal-processing pipeline.

The following libraries were used for image signal preprocessing and augmentation:

OpenCV was used for image manipulation and normalization, and DICOM was used for PNG conversion and processing intensities as two-dimensional signals.

Albumentations was used to apply stochastic augmentations to simulate acquisitional variations and rotation and translation as spatial signal transformations.

Pandas was used for managing patient metadata and datasets.

Scikit-learn was used to split training, validation, and testing sets and calculate performance metrics.

The experiments were conducted on a Kaggle Notebook equipped with two NVIDIA T4 GPUs (16 GB of VRAM each) and 52 GB of RAM. This system allows for rapid training and reproducible evaluation in accordance with CAD system standards.

### 3.2. Dataset and Preprocessing

#### 3.2.1. Dataset

The RSNA Breast Cancer Screening Mammography Dataset comprises 54,706 images from 33,552 patients, along with all associated metadata, such as age, side, view, and BI-RADS density. Each patient has craniocaudal (CC) and mediolateral oblique (MLO) views, providing complementary signals for analysis [41].

The RSNA dataset employed in the present investigation is publicly available and fully anonymized. Consequently, in compliance with institutional and ethical guidelines regarding the utilization of publicly available anonymized data, the approval of the Institutional Review Board (IRB) and informed consent were not required for this study.

#### 3.2.2. Preprocessing: Image Preprocessing

To optimize the quality and representativeness of the mammographic image for a CAD system, the following pipeline was applied:

1. Removal of missing values

The signal corresponding to the missing densities was removed, so the number of images decreased from 54,706 to 29,470.

2. Implant exclusion

To prevent artificial alterations of parenchymal appearance and density distribution, it was applied to exclude all images containing implants (*n* = 10,460). While this step enhances signal homogeneity and label reliability, it may reduce anatomical and acquisition variability and can potentially influence generalizability to implant-bearing patients, as indicated in Section 5.6.

3. Density filtering

Only categories B and C were retained, corresponding to 23,513 images, as they are most prone to visual ambiguity.

The acknowledged ambiguity and high inter-observer variability associated with the interpretation of BI-RADS categories B and C are the main factors behind this choice. In fact, these two elements made these two categories particularly relevant targets for automated, objective, and reproducible assessment based on deep learning.

4. Label encoding

For computational processing, the categorical labels were transformed into a numerical format, in accordance with the next encoding scheme: BI-RADS B → 0 and BI-RADS C → 1. It is to be noted that this encryption is specifically used for model training. It does not assume any ordinal relationship between the classes.

5. Patient-wise stratification

The dataset under consideration comprises 4796 unique patients associated with 23,513 images (2460 patients in class B and 2336 patients in class C).

Every patient is associated with several mammographic views, encompassing the left and right breasts as well as standard craniocaudal (CC) and mediolateral oblique (MLO) projections. A one-of-a-kind identifier was used for each patient to group images from the same patient and hence prevent data leakage between the training, validation, and testing sets.

This stratification simulates a practical clinical evaluation.

6. Normalization and enhancement

The original images are single-channel (grayscale). They were converted to 3-channel images using channel duplication to conform to the input requirements of the pre-trained CNN.

Training: Resize (380 × 380), Horizontal Flip, ShiftScaleRotate, Brightness/Contrast, and CoarseDropout.

Validation/Test: Resize + normalization (ImageNet stats).

It is important to note that the data augmentation was applied solely to the training set. The validation and test sets were kept unchanged.

These different steps enhance the robustness of the CAD system in the face of acquisition variability.

#### 3.2.3. Set Division

The dataset, as shown in Table 1, was divided into training, validation, and test sets to ensure efficient training and reliable evaluation. Furthermore, the class distribution remained balanced within each subset, helping to limit bias and ensure representative model performance.

### 3.3. Model Architecture: EfficientNet–UNet Hybrid Framework

**Figure 1:**
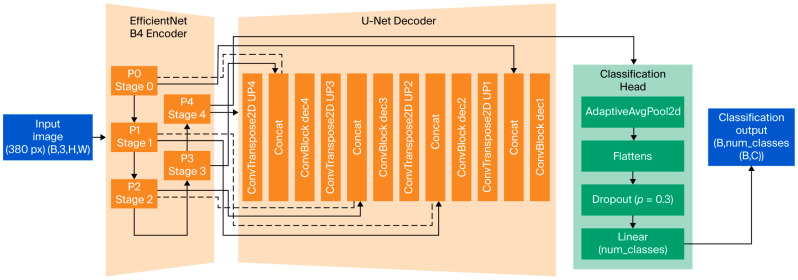
Architecture of the EfficientNet–UNet hybrid model.

The model at issue merges the multi-scale feature extraction potential of EfficientNet with the preservation of high-resolution details of U-Net skip connections, hence optimizing the mammographic image representation at various resolutions. The architecture is designed for its likely implementation in an entire CAD processing chain, including multi-view processing and interpretability. This architecture is exceptionally well fitted to the BI-RADS B vs. C classification, in which subtle differences in fibroglandular texture and spatial distribution need to be preserved. This limitation is frequently encountered in standard classification encoders, which increasingly lose fine spatial information. In addition, fine-grained structural details can be recovered by integrating U-Net skip connections, while robust hierarchical representations of features are provided by EfficientNet. This leads to a particularly effective complementary mechanism for modeling subtle density variations, as shown in Figure 1.

#### 3.3.1. Key Components

The EfficientNet Encoder, which extracts feature maps at different spatial scales (p0–p4), thus representing the signal at increasingly higher resolution.

The refinement of spatial features is achieved using the U-Net decoder by oversampling and progressively integrating the encoder feature maps via skip connections, which allows for the recovery of fine-grained structural specifications.

ConvBlock, which consists of two successive convolutions followed by batch normalization (BatchNorm) and ReLU activation to refine the learned representations.

Adaptive Upsampling, which combines transposed convolution (ConvTranspose2d) and bilinear interpolation to align feature maps at different scales.

The Classification Head, which consists of 2D adaptive average pooling, followed by a dropout layer and a linear layer, allowing it to capture the overall signal information. This classification head is appropriate for generating prone outputs for integration into a CAD system.

#### 3.3.2. Model Parameters

Encoder: We used the EfficientNet-B4 encoder, with feature extraction only (features_only = True) and pre-trained weights (pre-trained = True) for efficient transfer learning.

Input size: 380 × 380 × 3.

Decoder: The decoder consists of four stages, each combining a transposed convolution (ConvTranspose2d) and a convolutional block (ConvBlock). It ensures the local-global fusion of imaging information.

Classification head: This component reduces the 1792 input neurons to 2 output neurons, with a dropout of 0.3 to limit overfitting.

Total parameters: There are approximately 21.3 million parameters, i.e., 19.2 million for the encoder and 2.1 million for the decoder. This setting is compatible with a standard GPU deployment for a clinical CAD.

#### 3.3.3. Multi-View Fusion

Let $f_{CC}∊R^{d}$ and $f_{MLO}∊R^{d}$ refer to the feature representations extracted, respectively, from the craniocaudal (CC) and mediolateral oblique (MLO) views. The feature-level fusion is carried out using simple concatenation. It is expressed as

(1) $$f_{fusion}=\left[f_{CC};f_{MLO}\right]{∊R}^{2d}$$

This technique allows for the learning of joint multi-view representations for the characterization of breast density.

Unlike the decision-level fusion, which merges predictions after independent inference, feature-level concatenation maintains all inter-view dependencies and enables the network to directly learn inter-view correlations within the representation space.

The EfficientNet-B4 encoder extracts multi-scale hierarchical representations for each mammographic view. In contrast, the U-Net decoder serves as a spatial feature enhancement module. It is based on an encoder/decoder structure with hop connections (skip connections) to keep local spatial information.

Multi-view merging is carried out after the global average pooling (GAP) of each branch and prior to the classification head (dropout layers and fully connected layers). This should ensure that feature integration takes place within the latent representation space while preserving view-specific discriminating information.

### 3.4. Training

Optimizer: AdamW (LR encoder 1 × 10^−5^, LR decoder 1 × 10^−4^).

Scheduler: CosineAnnealingWarmRestarts (T_0 = 5).

Loss: CrossEntropyLoss with label smoothing 0.1 was used to stabilize learning and limit overfitting for redundant features.

Batch size: 16. Epochs: 50 (early-stopping patience = 10).

Multi-GPU: nn.DataParallel was used to accelerate training and guarantee reproducibility.

### 3.5. Test Data and Ground Truth

The test set included 2321 mammographic images that were subjected to patient-wise stratification to preserve the inter-patient variability and avoid any data leakage between training, validation, and testing sets.

Reference annotations were based on the BI-RADS B vs. C classifications that are provided in the dataset. These are considered as the primary reference for training and evaluating the model. A consent reading between the two radiologists served as the gold standard in case of disagreement. This was necessary to diminish the inter-observer variability and provide a stable reference for clinical comparison.

### 3.6. Statistical Analysis

To thoroughly assess the performance of our breast density classification pipeline, we deemed it appropriate to apply a multi-level approach that integrates standard signal-processing metrics, training dynamics analysis, and explainability tools to ensure our assessment was comprehensive and clinically oriented.

#### 3.6.1. Performance Metrics

Standard evaluation metrics, which are considered indicators of the quality of the mammographic image representation learned by the model, were calculated for binary classification. These metrics are given below.

Accuracy: This metric indicates the proportion of correctly classified mammograms.

Precision, Recall, and F1score: These metrics were computed per class to assess the model’s potential to accurately identify BI-RADS density categories B and C while minimizing the false positives and false negatives.

ROC-AUC: This metric, the area under the receiver operating characteristic curve, was used as a global measure of the model’s discriminative capacity through the multi-view input signal.

#### 3.6.2. Training Dynamics

Loss and accuracy curves for the entire training/validation phase were used to monitor the learning process, detect any overfitting or underfitting, and analyze the stability and convergence of the model.

Resource monitoring or tracking was performed. The average training time per epoch and GPU and CPU utilization were assessed. This process guaranteed the model’s practical feasibility for employment in standard systems.

#### 3.6.3. Multi-View Fusion and Image Preprocessing

The impact of multi-view fusion (CC+MLO) on classification performance was thoroughly assessed:

Each view is considered a partial representation of the breast signal.

The concatenation of features extracted at different scales represents a multi-view fusion. It improves the robustness of predictions and consistency between the two breasts of the same patient.

The metrics were calculated for each view separately and for the merged view to quantify the contribution of fusion to the overall performance.

#### 3.6.4. Additional Analysis

We also investigated the following:

The distribution of errors according to breast density and technical artifacts was determined.

The correlation between model performance and breast tissue complexity (heterogeneous vs. dispersed tissue) was examined to better understand the model’s limitations and optimize the CAD pipeline.

### 3.7. Explainability and Clinical Validation

Furthermore, the image regions contributing to model predictions in breast density classification were visualized by means of the Gradient-weighted Class Activation Mapping (Grad-CAM) technique. Moreover, two board-certified radiologists, including a co-author radiologist and an independent hospital-based professor of radiology, performed the qualitative evaluation. These two experts independently examined some representative Grad-CAM cases for the purpose of determining whether the highlighted regions were clinically relevant fibroglandular tissue patterns.

Any disagreement arising between the two readers was discussed to reach a consent-based solution and thus achieve a radiological consensus. This evaluation was primarily aimed at assessing the anatomical plausibility of the model’s attention maps and at giving some qualitative clinical insight into its decision-making process.

The inter-reader evaluation evidenced that Grad-CAM activations were, for the most part, localized in regions of fibroglandular tissue associated with BI-RADS C density. This confirms a good anatomical agreement with the radiological interpretation. It should be noted that no consistent activation was noticed in non-anatomical regions such as background artifacts or unrelated structures.

## 4. Results

### 4.1. Overall Model Performance

The proposed EfficientNet–UNet hybrid model was evaluated on the test set. This set consists of 2321 images from 479 unique patients. The aim was to perform binary classification of breast densities based on BI-RADS B and C.

For the sake of comparison, two other architectures, namely, ResNet-50 and Vision Transformer, were also employed. The results are shown in Table 2.

The model achieved an accuracy of 87% and an AUC of 94.40%, confirming its ability to effectively distinguish between intermediate densities B and C. Compared to classical CNN architectures or Transformers, the model we developed offers a competitive balance between precision and calculation time, making it well suited to a CAD clinical application.

In addition, qualitative evaluation of model explanations demonstrated strong consistency with expert radiological interpretation based on independent dual-reader assessment of Grad-CAM visualizations.

Most importantly, the performance of the proposed model (AUC = 94.40%) is within the inter-radiologist variability range that is widely documented in the literature for breast density assessment, specifically for the clinically complex BI-RADS B vs. C boundary. This indicates that the suggested model presents a diagnostic behavior that is similar to that provided by expert radiologists confronted with ambiguous intermediate-density cases.

### 4.2. Impact of Multi-View Learning (CC+MLO)

The combined use of CC and MLO views contributes to improving the model performance and strengthening the coherence of predictions between the left and right breasts.

The fine-grained details and overall contextual information within each individual view are simultaneously captured using the multi-scale feature learning approach. At the same time, the multi-view fusion is achieved solely by means of feature-level concatenation of CC and MLO representations before classification.

This unequivocal distinction between intra-view representation learning and inter-view fusion helps to improve the way to deal with borderline B vs. C cases by exploiting complementary anatomical information from the two projections.

It can therefore be asserted that, overall, this design enhances the classification accuracy and corroborates the clinically meaningful interpretation in breast density evaluation on the RSNA dataset.

McNemar and DeLong tests were employed to carry out a formal statistical comparison between the suggested framework and the reference multi-view configuration. It should be emphasized that although the proposed model achieved the best observed performance in terms of accuracy, AUC, F1 score, and sensitivity, the improvements observed were relatively modest and ought to be interpreted within the scope of the comparisons being evaluated. Although these differences are statistically significant compared to the reference values evaluated (McNemar: *p* = 0.0024; DeLong: *p* = 0.0352), they remain limited between intimately linked configurations. Nevertheless, the proposed framework exhibited fairly stable performance according to the evaluation indicators. This was corroborated by an explainability analysis with radiological validation conducted by experts. This confirms its potential clinical applicability and consistency.

### 4.3. ROC Curves, Confusion Matrix, and Recall

**Figure 2:**
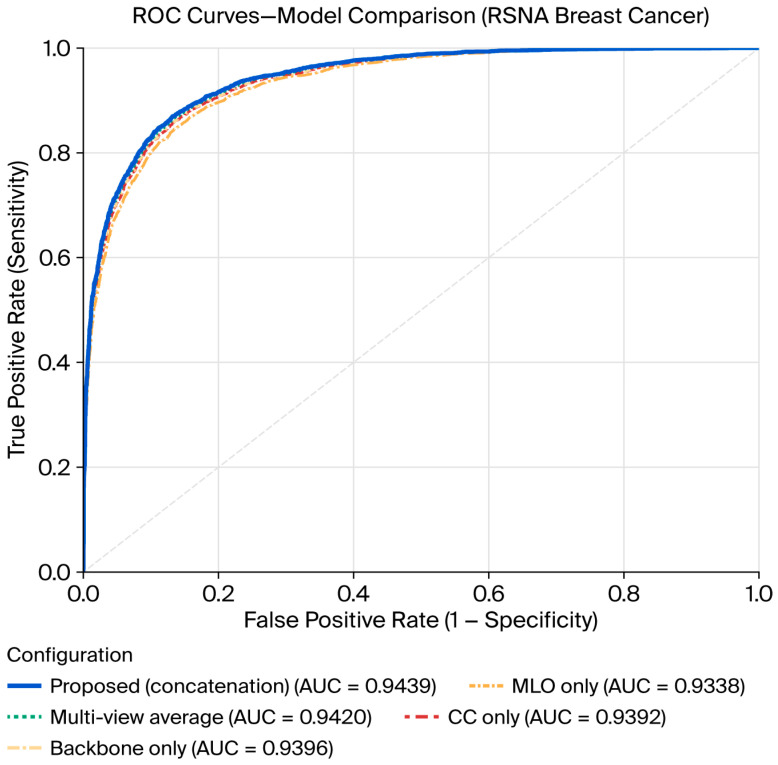
Receiver operating characteristic (ROC) curves for BI-RADS B versus C classification, demonstrating the high discriminative performance of the proposed model.

**Figure 3:**
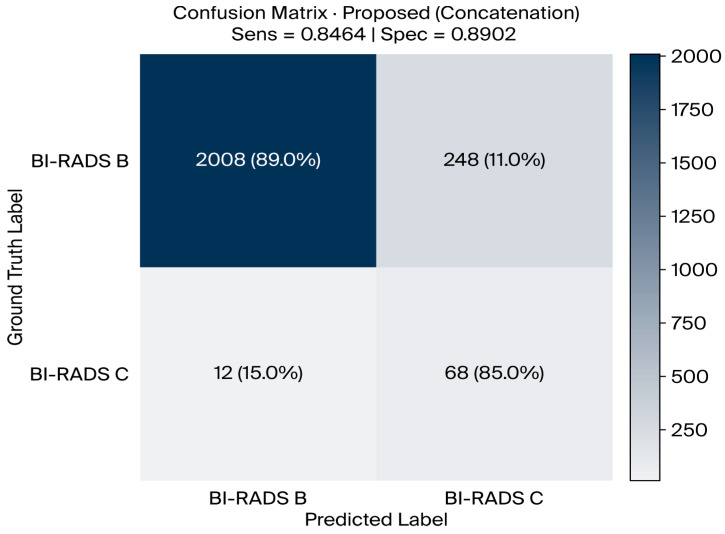
Confusion matrix illustrating classification performance between BI-RADS B and C classes, showing balanced prediction distribution.

The ROC curve in Figure 2 shows an AUC of 94.40%, confirming excellent discrimination between B and C. Figure 3 presents the confusion matrix, which confirms there is a balanced distribution between the classes. This is essential for a reliable CAD.

The data reported in Table 3 show coherent class-wise performance through BI-RADS B and C categories. This is clinically important for a dependable assessment of intermediate breast densities.

The model attained balanced class-wise performance through both BI-RADS categories, with a moderately higher recall for class B while preserving a high sensitivity for class C, which remains clinically important for identifying dense breasts. This feature is excellent for reliably detecting and assessing dense breast tissue.

### 4.4. Learning Dynamics

**Figure 4:**
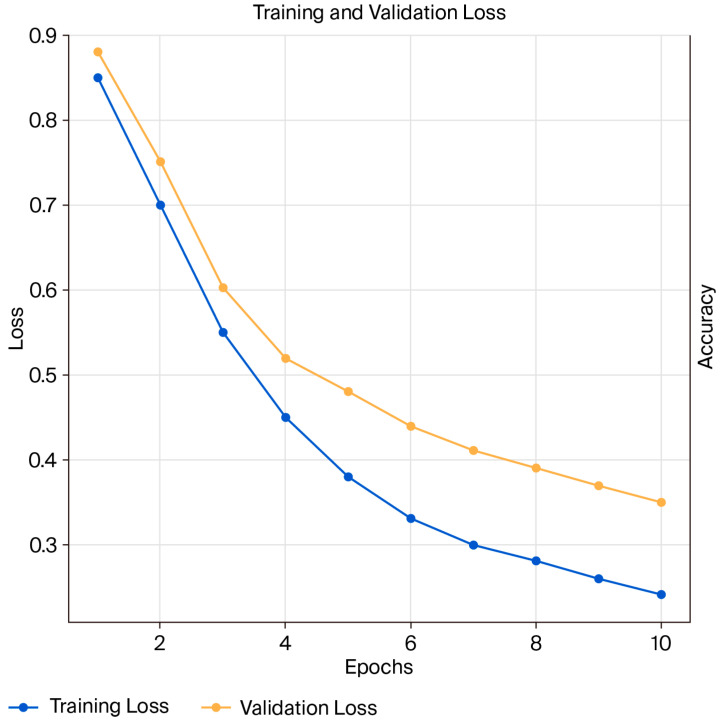
Training and validation loss curves demonstrating stable convergence of the proposed model during training.

**Figure 5:**
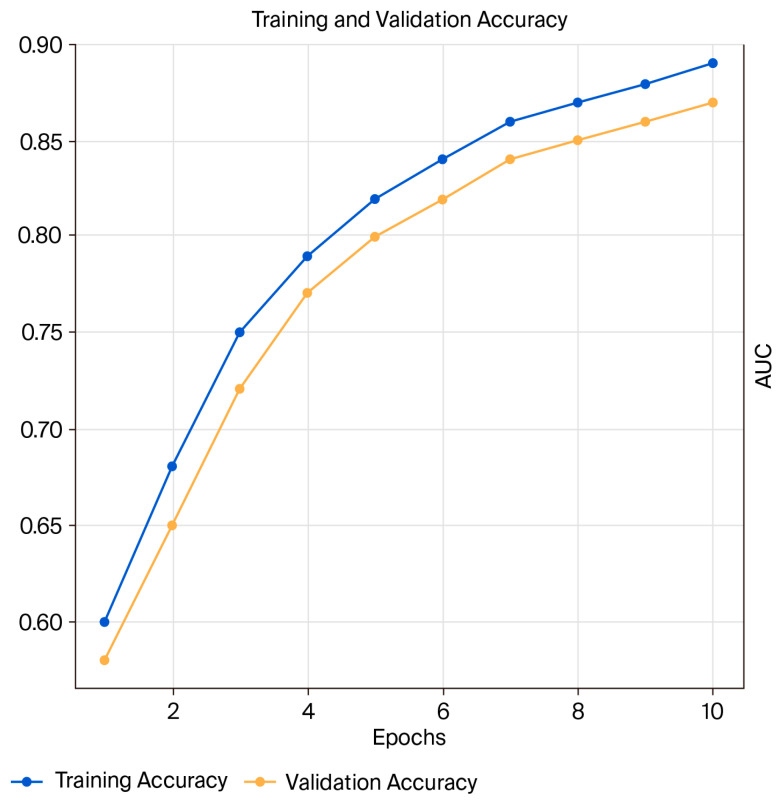
Training and validation accuracy curves showing consistent learning behavior and reduced overfitting.

**Figure 6:**
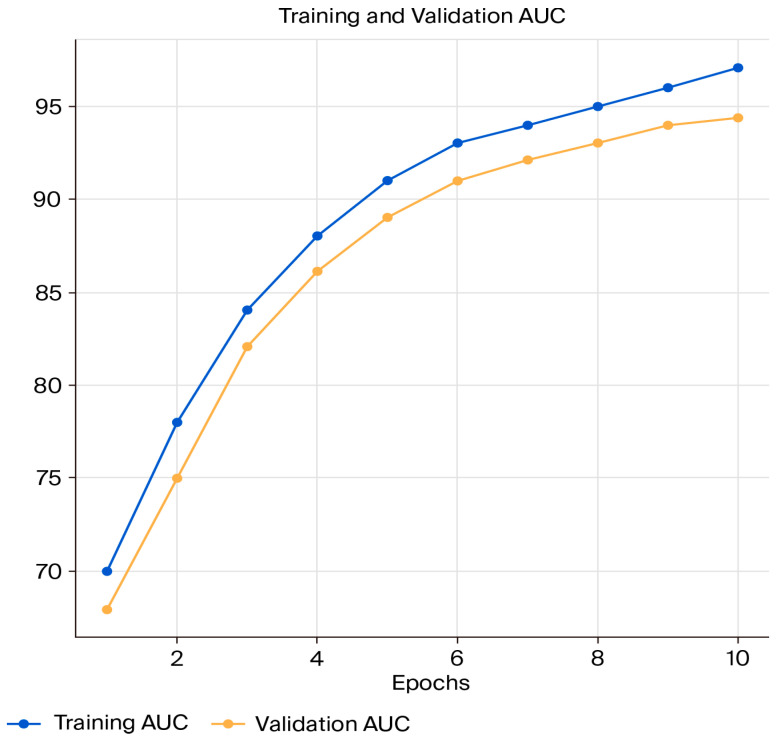
Training and validation AUC curves illustrating progressive improvement and stable model performance.

Performance monitoring during the training process (Figure 4, Figure 5 and Figure 6) is summarized below. The total training duration was 207 min.

Early stopping was applied with a patience period of 10 epochs while monitoring the validation loss as the main metric. Training continued until no validation loss improvement was noticed over 10 successive epochs. The highest-performing model was chosen based on the lowest validation loss on the validation set.

### 4.5. Grad-CAM Visualization

The Grad-CAM technique allows for the identification of parenchymal areas that contribute most to the model’s decision.

**Figure 7:**
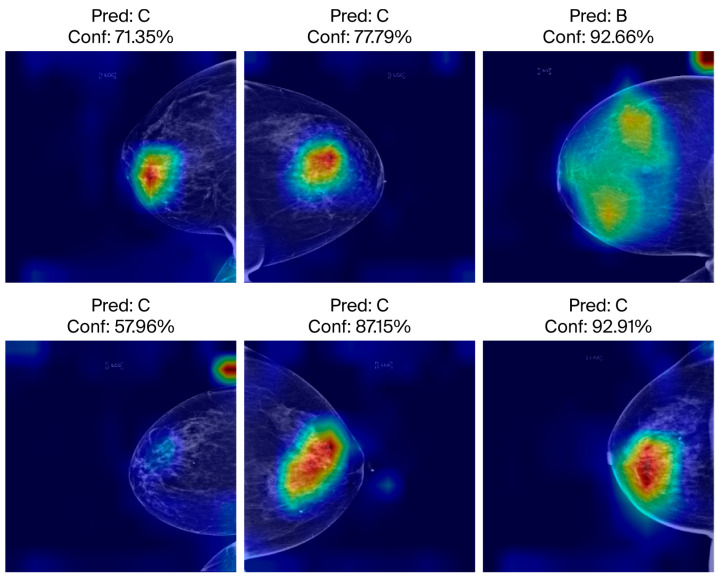
Grad-CAM visualizations highlighting regions of interest in mammographic images, focusing on fibroglandular tissue relevant to breast density classification.

The activation maps in Figure 7 confirm that the model concentrates primarily on relevant regions, not artifacts, thus aiding clinical interpretability.

Thus, a detailed error analysis and radiological validation were conducted to more accurately assess the interpretability of the model in ambiguous cases. These are presented in the following section.

### 4.6. Error Analysis and Clinical Validation

#### 4.6.1. Failure Analysis (False Positives and False Negatives)

To provide a clinically interpretable assessment of the model’s limitations, it was deemed necessary to carry out a systematic error analysis of representative cases of false positives (FP) and false negatives (FN) extracted from the test set.

False positives (BI-RADS B → C), mainly associated with diffuse, heterogeneous, or low-contrast density patterns, were intrinsically challenging to discern.

**Figure 8:**
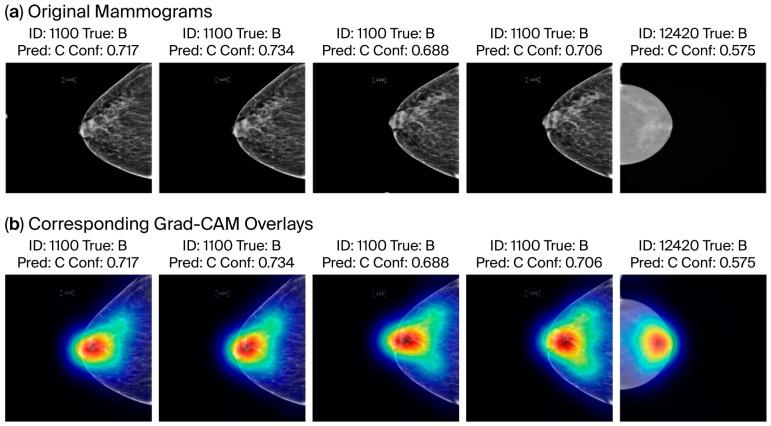
Representative false positive cases (true BI-RADS B, predicted BI-RADS C): (**a**) original mammograms; (**b**) corresponding Grad-CAM overlays highlighting the image regions that contributed to the overestimation of breast density by the proposed model.

**Figure 9:**
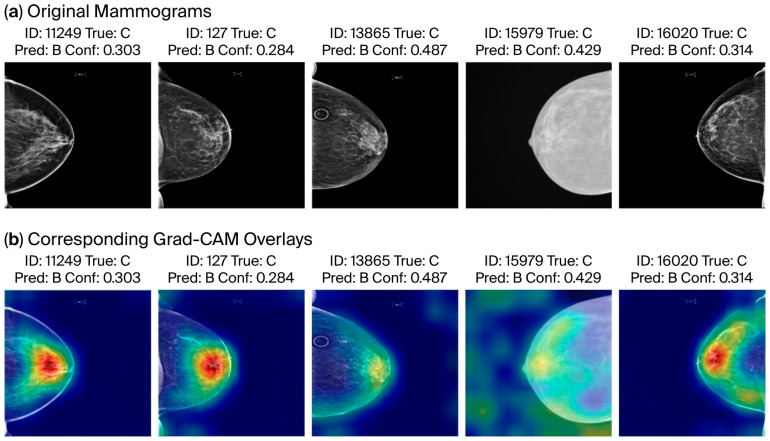
Representative false negative cases (true BI-RADS C, predicted BI-RADS B): (**a**) original mammograms; (**b**) corresponding Grad-CAM overlays highlighting the image regions that contributed to the underestimation of breast density by the proposed model.

Figure 8 (false positives) and Figure 9 (false negatives) present representative examples of these failure modes. In both figures, the top row shows the original mammograms, while the bottom row displays the corresponding Grad-CAM overlays. These visualizations highlight the image regions that contributed most strongly to the model’s predictions.

Table 4 and Table 5 provide a structured quantitative and qualitative analysis of FP and FN cases, summarizing radiological results, Grad-CAM activation maps, and clinical explanations for each classification error.

Besides the standard FP/FN cases, additional challenging cases in dense and heterogeneous breast tissue were analyzed in more detail to gain a better understanding of the model’s behavior under difficult and complex imaging conditions.

**Figure 10:**
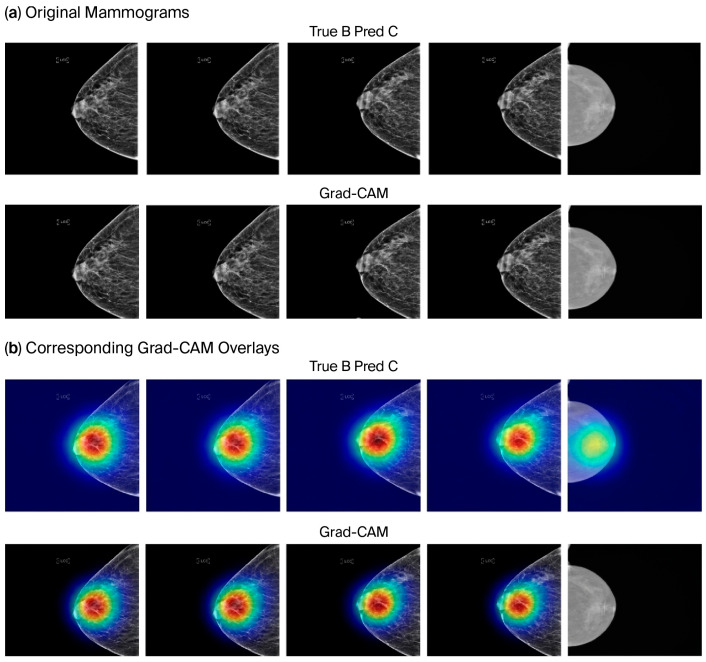
Representative challenging false positive cases with complex and heterogeneous fibroglandular architecture (true BI-RADS B, predicted BI-RADS C): (**a**) original mammograms; (**b**) corresponding Grad-CAM overlays highlighting the image regions that contributed to the model’s prediction in challenging cases with complex and heterogeneous fibroglandular tissue patterns.

Challenging false positive cases with complex and heterogeneous fibroglandular architecture are distinguished by subtle architectural distortions, overlapping fibroglandular structures, and localized high-density regions. These cases are particularly difficult to interpret because of ambiguous radiological boundaries, as illustrated in Figure 10, where partially aligned or regionally diffuse Grad-CAM activations are observed.

**Figure 11:**
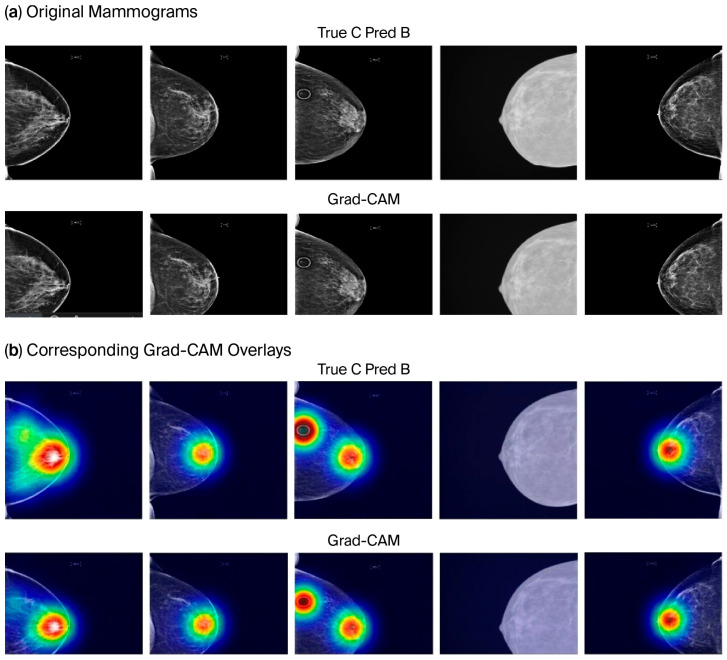
Representative challenging false negative cases in dense and heterogeneous breast tissue (true BI-RADS C, predicted BI-RADS B): (**a**) original mammograms; (**b**) corresponding Grad-CAM overlays highlighting weak or diffuse activation patterns associated with the model’s underestimation of breast density in challenging dense and heterogeneous breast tissue.

Likewise, challenging false negative cases in dense and heterogeneous breast tissue frequently entail dense breast tissue, imaging artifacts, and reduced lesion visibility. Figure 11 clearly indicates that these cases are related to weak or diffuse Grad-CAM activations, suggesting decreased model sensitivity in low-contrast or highly complex anatomical contexts.

Unlike the representative false-positive cases shown in Figure 8, these challenging cases exhibit more extensive and heterogeneous fibroglandular tissue distributions, resulting in diffuse activation patterns and increased ambiguity near the BI-RADS B vs. C classification boundary.

Unlike the representative false-negative cases shown in Figure 9, these challenging cases exhibit more extensive dense tissue, weaker Grad-CAM activations, and greater anatomical complexity, making accurate breast density estimation more difficult.

#### 4.6.2. Expert Validation of Grad-CAM and Explainability Assessment

The Grad-CAM activation maps were evaluated by two board-certified radiologists. One is a co-author, while the other is a completely independent hospital expert who was not involved in the development, training, or analysis of the model. This evaluation aimed primarily to assess the concordance between the model’s explanations and clinically applicable radiological patterns.

One of the two radiologists participated in the design of this study, while the second was completely independent, as he played no role in the development and training of the model or in data analysis.

The 100 different cases used for the Grad-CAM evaluation were selected at random from the independent test set. Furthermore, the sampling was carried out in a class-stratified manner to maintain the BI-RADS B vs. C distribution, without any selection based on model predictions or confidence scores.

The inter-observer agreement between the two radiologists was quite significant, with a Cohen’s kappa coefficient κ equal to 0.82, suggesting a coherent interpretation of Grad-CAM visual patterns.

Agreement between the model explanations and radiological assessment reached the value κ = 0.85, indicating a usually coherent correspondence between the highlighted regions and clinically pertinent fibroglandular structures in BI-RADS B and C cases.

The results of the evaluation (*N* = 100) are summarized in Table 6, in which the percentages correspond directly to the number of cases (e.g., 54 fully aligned cases, 31 partially aligned cases, and 15 misaligned cases).

It may therefore be stated that the evaluation protocol was globally designed to diminish the selection bias through the use of an independent test subset with random class-stratified sampling. We also acknowledge that the participation of a co-authoring radiologist may bring out a residual confirmation bias risk, which can be considered a limitation of this study.

For the sake of clarity, given that *N* = 100, the percentages shown correspond directly to the number of cases. The consensus assessment resulted in 54 perfectly aligned cases, 31 partially aligned cases, and 15 non-aligned cases, due to the agreement between the two readers who employed a predefined consensus procedure.

The discrepancies between the individual assessments of readers were resolved during this consensus procedure.

The evaluation protocol was globally designed to minimize selection and confirmation bias, using a randomly sampled, class-stratified independent test subset, and involving an external radiologist who did not contribute to the development or analysis of the model.

#### 4.6.3. Clinical Interpretation and Model Reliability

The combination of quantitative and qualitative analyses proves that the proposed system generates clinically consistent predictions, based on anatomically meaningful structures.

The agreement between the Grad-CAM activation maps and radiological consensus was assessed using Cohen’s weighted kappa (Table 7) to quantify the explainability performance. The model achieved substantial agreement with the expert consensus (κ = 0.85), with an exact agreement of 87%, indicating that performance in harmony with the inter-observer variability between radiologists (κ = 0.82).

The values obtained are in good agreement with the levels of inter-observer agreement previously reported in breast density assessment studies, in which the Cohen’s kappa coefficient generally starts from moderate to substantial agreement, depending on the complexity of the task. In particular, the BI-RADS density classification is known to show inherent variability between radiologists, particularly in borderline cases between categories B and C, which enhances the clinical pertinence of the observed agreement.

#### 4.6.4. Summary

It can therefore be concluded that, overall, the proposed framework exhibits robust, clinically consistent, and anatomically sound behavior for breast density classification, especially in the exceptionally complex BI-RADS B vs. C transition zone. The integration of multi-view deep learning with explainability (Grad-CAM) guarantees that predictions and error cases are based on anatomically relevant features. Crucially, the model achieves levels of agreement similar to inter-observer variability in breast density assessment, thus strengthening its potential as a reliable clinical decision support tool.

The expert review, though limited to 100 cases, provides valuable qualitative validation of the model’s explainability and clinical relevance. Forthcoming work will focus on validating the proposed framework on larger, multicenter datasets and expanding the review to other clinical scenarios, inclusive of prospective clinical validation and incorporation into actual diagnostic workflows.

These results corroborate the substantial role of the proposed system as a decision support tool likely to diminish the inter-observer variability and enhance consistency in the assessment of borderline breast density.

### 4.7. Comparison with State-of-the-Art Models

To situate our work within the corresponding scientific context and highlight its contribution, we decided that it was essential to compare our EfficientNet–UNet hybrid pipeline with recently developed mammography-based breast density classification methods. The main performance indicators, including published results as well as our own experience with the large, diverse, and clinically relevant RSNA dataset, are all summarized in Table 8.

Most existing methods rely on single-view inputs or simple averaging strategies for multi-view merging, restricting their potential to capture complex inter-view correlations. Some methods, such as TwoViewDensityNet developed by [27], demonstrate very high performance on multi-class classification tasks (4-class BI-RADS). However, it is important to note that these results cannot be directly compared to B vs. C classification, which is generally considered the most clinically ambiguous and challenging boundary. Furthermore, it should be emphasized that the datasets used in these studies are very different. For instance, DDSM and IN-breast are smaller and more homogeneous. In addition, these methods typically employ an image-wise protocol, which may lead to optimistic performance estimates when evaluated patient-wise. Conversely, our approach focuses specifically on the B vs. C task and uses a patient-wise split on the large RSNA dataset (23,513 images, 4796 patients). This provides a much more realistic assessment of the model’s generalization capacity. This configuration highlights the clinical difficulty of the task and the robustness of our approach in the face of cases representative of real clinical practice.

In contrast, our EfficientNet–UNet hybrid framework unambiguously concatenates feature representations from CC and MLO views, capturing both local and global tissue patterns. This pattern not only enhances classification performance but also produces interpretable outputs via Grad-CAM, hence corroborating clinical decision-making. Critically, all model outputs were calibrated, guaranteeing that the predicted probabilities are reliable and suitable for clinical decision support.

A contextual and descriptive comparison with previous studies is provided in Table 8. This comparison is not intended to establish benchmarks or for the ranking of performance. Given the differences between the datasets, class definitions, and evaluation protocols (image-wise or patient-wise), the results presented should be interpreted with great caution. They do not allow for a strict ranking of the methods. This comparison, which is essentially qualitative, aims to highlight methodological trends observed in the literature.

The performance reported in previous studies should be approached carefully, as image-by-image evaluation protocols and decreased inter-subject variability may result in optimistic estimates that do not reflect real-life conditions of clinical deployment. This phenomenon has been extensively reported in the medical imaging literature, in which evaluation protocols and dataset characteristics may bring in systematic bias, which results in model performance overestimation and lower external validity in practical settings [43,44,45].

It should be noted, however, that the model proposed here is evaluated using a patient-based protocol on a large real-world dataset (RSNA). This provides a more realistic and clinically relevant assessment of generalizability in screening settings.

The performance observed with this protocol reflects higher task complexity and lower information leakage instead of inherent limitations of the model.

Table 8 shows considerable methodological heterogeneity among previous studies, especially regarding the nature of datasets and evaluation protocols. Furthermore, because methods evaluated on selected datasets (e.g., INbreast and DDSM) generally benefit from controlled acquisition parameters and image-wise evaluation, performance indicators and metrics may be optimistically biased. On the other hand, patient-based evaluation on RSNA offers a much more rigorous and clinically realistic framework, leading to smaller but more reliable generalization performance estimates.

Therefore, the performance differences observed between the different studies ought to be explained and elucidated within the context of their respective experimental conditions rather than as direct comparisons of the models’ capabilities.

They should rather be scrutinized in relation to robustness and clinical relevance under authentic screening conditions.

Although convolutional neural networks, such as the ResNet-based architectures, offer excellent mammographic analysis performance, they remain weak in expressly modeling view dependencies and in providing the interpretability of results. Likewise, current multi-view combination strategies often depend on basic aggregation mechanisms (e.g., averaging or independent processing of views), which may not take full advantage of the complementary information between craniocaudal (CC) and mediolateral oblique (MLO) projections.

In this context, the proposed framework (EfficientNet-B4 + U-Net) helps address these limitations by merging CC and MLO views at the feature level, thus enabling richer and more discriminative multi-view representations. In addition, Grad-CAM is integrated to guarantee a posteriori interpretability of the model’s predictions, thereby improving clinical transparency.

This study focuses essentially on the BI-RADS B vs. C boundary, which is viewed as a clinically pertinent but intrinsically ambiguous classification situation that is distinguished by significant inter-observer variability. This makes it an exceptionally suitable framework for assessing AI-aided decision support systems (AI-DSS) under conditions of practical screening.

On the whole, the proposed framework is configured as a clinical decision support system rather than an autonomous diagnostic tool. Though the results exhibit solid performance within a practical evaluation protocol, validation remains restricted to a single cohort. Therefore, prospective multicenter clinical studies should be included in future work to evaluate the robustness and generalizability of the model before its clinical deployment, with greater precision.

### 4.8. Qualitative Summary

**Figure 12:**
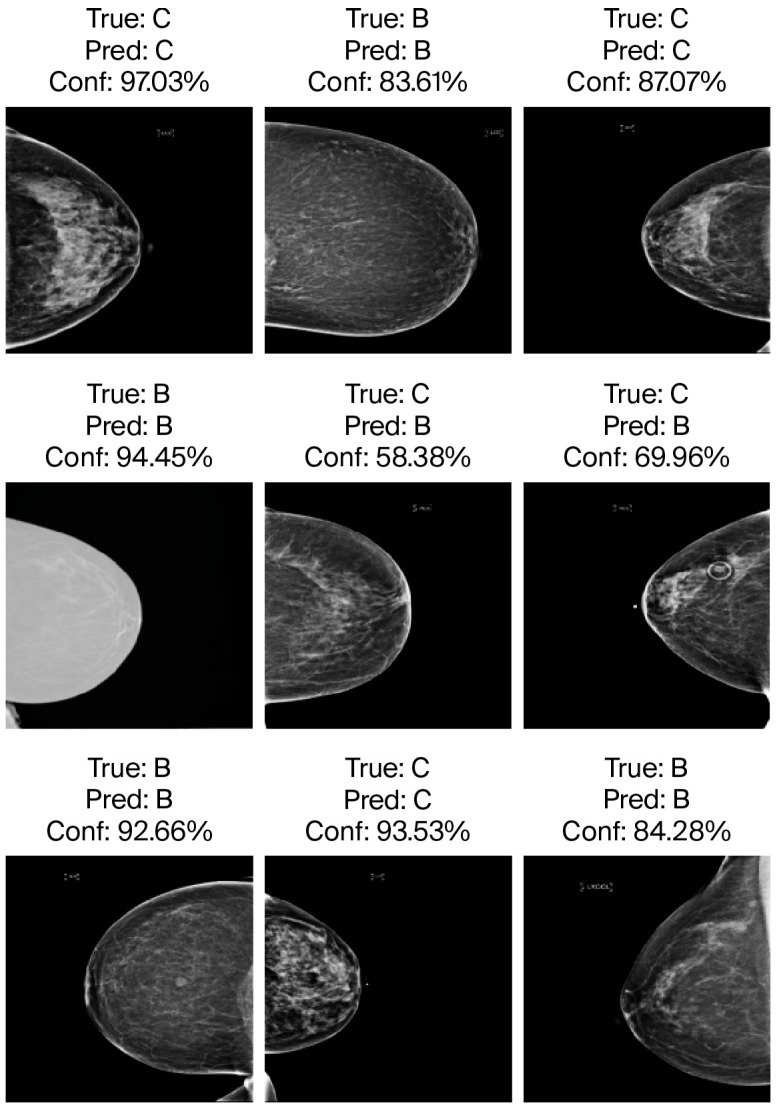
Representative examples of correct and incorrect predictions. The parenchymal areas used by the model are clearly shown.

Figure 12 illustrates some representative examples of correct and incorrect predictions.

Correct predictions: These are cases where the model accurately identified breast density patterns, with B → dispersed fibroglandular tissue, and C → heterogeneous structures.

Incorrect predictions: There are borderline cases of B vs. C or technical artifacts (blurring, poor acquisition, and insufficient contrast).

Multi-view and multi-scale exploitation: The model effectively uses CC and MLO projections and multi-scale features, guaranteeing reliable and interpretable classification even for complex cases.

### 4.9. Ablation, Performance, and Statistical Analysis

A patient-wise ablation study was carried out by evaluating seven configurations to assess the contribution of each architectural component:

- CC-only;
- MLO-only;
- Multi-average (probability-level fusion);
- Backbone-only;
- Multi-view feature-level concatenation without U-Net refinement;
- EfficientNet + U-Net (CC-only, no multi-view fusion);
- Proposed full model combining CC–MLO feature-level fusion with U-Net-based refinement.

**Figure 13:**
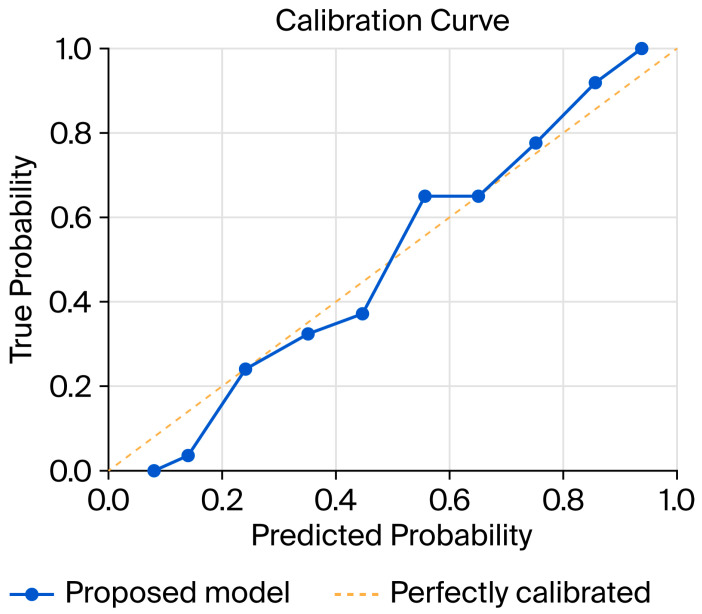
Calibration curve for predicted probabilities versus observed outcomes for BI-RADS B vs. C classification. The greater the proximity of the curve to the diagonal line, the better the calibration.

Identical preprocessing and optimization settings were employed for the training of all models. Table 9 summarizes the results obtained, including 95% confidence intervals. Besides the standard performance metrics (Accuracy, AUC, and Sensitivity), model calibration was also evaluated to guarantee reliable probability estimates. This yielded some insights into the discrimination and trustworthiness of the predicted probabilities, as depicted in Figure 13.

McNemar’s test for pairwise classification performance and DeLong’s test for comparison of areas under the curve (AUC) were employed to carry out formal statistical comparisons. Statistically significant improvements over the evaluated baselines, including the multi-mean fusion strategy (McNemar *p* = 0.0024; DeLong *p* = 0.0352), were supported by the proposed model. Furthermore, the robustness of the results was confirmed using bootstrap analysis (2000 resamples), with narrow confidence intervals for all metrics. While the absolute performance gains are somewhat small, the statistical analyses suggest that the proposed model consistently surpassed all competing fusion strategies.

The single-view models exhibit moderate discrimination, suggesting that CC and MLO views provide complementary density-related information.

The single-view reference methods were systematically surpassed by the feature-level fusion, which corroborates the advantage of using the joint multi-view representation learning framework.

Multi-average fusion enhances the AUC and sensitivity, emphasizing the advantage of combining views, although spatial interactions between features are not preserved.

Performance has already been improved compared with the backbone-only configuration by adding multi-view feature-level fusion (concatenation) without U-Net refinement. This indicates that feature-level fusion (concatenation)significantly contributes to discrimination performance. A further, more modest enhancement was thus achieved following the subsequent integration of the U-Net refinement module, suggesting that spatial refinement can offer additional advantages for modeling subtle density-related structures.

#### 4.9.1. Performance Summary

Compared with the backbone-only configuration, the proposed feature-level fusion (concatenation), with U-Net refinement, not only improves accuracy (0.870, 95% CI: 0.852–0.888 vs. 0.864, 95% CI: 0.846–0.882) and AUC (0.944, 95% CI: 0.926–0.962 vs. 0.940, 95% CI: 0.922–0.958) but also achieves the highest accuracy and AUC among all evaluated configurations, while maintaining competitive sensitivity (0.864, 95% CI: 0.846–0.882).

#### 4.9.2. Interpretation and Balance

Although some confidence intervals partially coincide across competing models, formal statistical testing indicated significant differences favoring the proposed framework. Based on these findings, the observed improvements should not be interpreted solely as performance trends but as improvements supported by statistical evidence.

#### 4.9.3. Final Evaluation

The proposed model showed robust performance (Accuracy: 0.870, 95% CI: 0.852–0.888; AUC: 0.944, 95% CI: 0.926–0.962; Sensitivity: 0.864, 95% CI: 0.846–0.882). In addition, the predicted probabilities are well calibrated (Brier score = 0.12; Figure 13), confirming the reliability of the model outputs. Calibration assessment is critical in decision support systems. Furthermore, reliable probability estimates are indispensable for clinical interpretation and risk communication.

#### 4.9.4. Conclusion

The previous results suggest that multi-view feature-level fusion with localized U-Net refinement contributes to competitive BI-RADS B vs. C discrimination while offering authentic probability estimates appropriate for clinical applications.

## 5. Discussion

Assessing breast density is a clinically relevant but complex task because of the inter-observer variability and subtle radiographic differences between adjacent BI-RADS categories, specifically at the B vs. C transition, a particularly ambiguous region in clinical practice. This study primarily focuses on this diagnostic boundary and examines a structured, multi-view deep learning framework in place of a comprehensive multi-class classification.

### 5.1. Clinical Interpretation of the Findings

Integrating CC and MLO views at the feature level significantly enhances classification performance compared to single-view approaches. While CC-only and MLO-only models achieved moderate discriminative capability, multi-view fusion showed slightly higher observed performance, hence corroborating the complementary nature of the two projections.

The feature-level concatenation approach proposed here offered the best accuracy and area under the curve (AUC). In contrast, the averaging fusion achieved a higher F1 score and sensitivity, depending on the protocol evaluated per patient. These outcomes suggest a complementary relationship between the two fusion strategies.

In addition, the previously mentioned findings indicate that the preservation of viewpoint-specific representations prior to classification could contribute to retaining subtle structural density patterns that would otherwise be attenuated by decision-level averaging, although the observed differences between merging strategies (fusion strategies) are quite small and should therefore be interpreted with great caution. Furthermore, the model’s results were adjusted to ensure reliable probability estimates that are appropriate for clinical decision support. The results presented in Table 8 reveal a high discriminatory potential for the BI-RADS B vs. C classification task, which is particularly complex in clinical settings. The significance of AI-based decision support systems is fundamentally obvious in confusing clinical contexts, such as the BI-RADS B vs. C classification, in which the inter-observer variability between radiologists is generally quite large.

Inter-observer agreement for BI-RADS breast density has been reported to vary across clinical settings, ranging from moderate agreement among community radiologists (κ = 0.58–0.70) [46] to substantial agreement (κ = 0.60–0.80) [46,47] and reaching higher levels in expert-controlled studies (κ up to approximately 0.85–0.90) [46,47]. In this regard, the proposed framework achieved a Cohen’s kappa of 0.85, suggesting performance comparable to expert-level agreement.

### 5.2. Hybrid Architecture Rationale

In this work, we propose a hybrid architecture integrating EfficientNet-B4, used as a feature extractor, with a U-Net-based refinement module. EfficientNet-B4 captures global hierarchical mammographic feature representations, while the U-Net decoder is especially utilized as a spatial feature refinement block for the enhancement of fine-grained texture information via skip connections. This model is especially convenient for distinguishing the subtle parenchymal differences between BI-RADS breast density categories B and C.

It is essential to underscore that the U-Net module is not employed for segmentation or pixel-by-pixel prediction. Furthermore, this module cannot be used for the generation of spatial masks or reconstructed outputs. It acts solely as an intermediate feature transformation step within the classification pipeline, aiming to enhance the quality of spatial representations prior to overall aggregation.

The decoder produces refined feature maps that preserve the spatial structure of the encoded representations. These feature maps are then clustered using global average pooling. This results in a compact feature vector that is passed to the fully connected final classification layer. This ensures that the task remains essentially a global image-level classification issue, while taking advantage of enhanced representations of spatial features.

The skip connections between EfficientNet-B4 encoder layers and the U-Net decoder allow the combination of high-level semantic features and low-level textural information. This fusion is extremely advantageous in mammography since the discriminating indices are distributed between both local fibroglandular structures and global breast parenchymal patterns.

It should be noted that U-Net-based refinement functions independently of the multi-view fusion mechanism. Furthermore, no cross-view interaction is conducted within this module. The multi-view integration is handled separately at the feature level by the concatenation of craniocaudal (CC) and mediolateral oblique (MLO) features. This ensures a manifest dissociation between intra-view spatial refinement and inter-view feature merging (multi-view feature fusion).

In contrast to former EfficientNet–UNet architectures, initially designed for segmentation or dense prediction tasks, the model presented here reutilizes the U-Net decoder as a classification-oriented feature enhancement module, with no pixel-level supervision or reconstruction loss.

The proposed pattern is based on the assumption that preserving and enhancing intermediate spatial representations before global pooling can help optimize discrimination in clinically ambiguous cases, specifically at the boundary between BI-RADS categories B and C. Moreover, the architecture remains completely compatible with classical classification frameworks based on convolutional neural networks (CNNs) while inducing minimal additional computational complexity. The contribution of this architecture is assessed in more detail in the extended ablation study that is discussed in Section 4.9.

### 5.3. Comparison with Prior Work

Several deep learning frameworks, including CNNs and hybrid CNN–Transformer architectures, have been developed for breast-imaging analysis. However, most studies have primarily dealt with lesion detection or segmentation tasks rather than structured multi-view breast density classification.

In the context of mammography-specific research, while multi-view approaches such as the TwoViewDensityNet method [27] incorporate dual projections (CC and MLO), they also generally rely on decision-level averaging or independent feature extraction streams without expressly modeling inter-view feature interactions. These strategies limit the capacity to capture complementary structural patterns between projections.

On the other hand, our framework performs explicit multi-view fusion at the feature level, allowing for the simultaneous modeling of local parenchymal textures and the overall distribution of breast tissue. It is worth noting that previous methods have not explicitly addressed this structured integration of multiple views, coupled with interpretability via Grad-CAM, as confirmed and demonstrated by an experienced radiologist.

Though some prior studies, such as that conducted by Busaleh et al. [27] on the TwoViewDensityNet method, report very high AUCs on multi-class tasks (4 BI-RADS classes), these results cannot be directly compared to the B vs. C classification, which represents the most ambiguous and difficult clinical boundary. In addition, previous studies have frequently employed smaller or selected datasets, such as DDSM and IN-breast, whereas our evaluation is based on the large RSNA dataset with a patient-wise split, which better reflects real clinical practice.

Although several reference methods have reported greater accuracy on small, selected datasets, our patient-wise assessment of the large-scale RSNA dataset exhibits higher quality clinical reliability and generalizability, highlighting the compromise between apparent accuracy and robustness.

It is worth noting that any straightforward numerical comparison between studies should be explained with great caution due to differences between datasets (DDSM, INbreast, RSNA), assessment protocols (image-wise vs. patient-wise), and task definitions (multi-class vs. binary classification). Consequently, Table 8 provides a qualitative rather than strictly quantitative comparison. Furthermore, the performance differences between image-wise and patient-wise evaluation protocols introduce a known systematic discrepancy, which inherently limits any immediate comparison. The suggested model is assessed according to a patient-based protocol, which is more clinically realistic and generally performs less well than image-based evaluation. This partly explains the differences observed with previous studies.

It is noteworthy that the proposed hybrid model (EfficientNet–UNet) clearly concatenates CC and MLO features, thus capturing the correlations between views that single-view or simple averaging methods cannot exploit. Moreover, this multi-view integration, combined with interpretability using Grad-CAM, promotes clinically relevant decision-making while preserving competitive predictive performance.

This approach contributes to diminishing the inter-observer variability. It also offers a highly dependable decision-making instrument for radiologists, thereby enhancing the consistency and accuracy of breast density assessment in the course of routine mammographic screening. All these features contribute to improving performance and reliability, as clearly indicated in Table 8.

Furthermore, new developments in breast cancer diagnosis have examined hybrid and transformer-based learning approaches. For example, the ETECADx framework introduces an Ensemble Self-Attention Transformer Encoder (often termed ETE or ViT-based architectures) from full-field digital breast radiographic images [48]. It demonstrates a high potential for capturing long-range dependencies in medical imaging.

Likewise, a hybrid explainable federated vision transformer framework has been designed for breast cancer prediction through the integration of imaging data with patient-specific risk factors [49]. This approach underscores the prospects of merging privacy-respecting learning and interpretability in clinical decision support systems.

These methods demonstrate an increasing trend for explainable, transformer-based AI architectures for the analysis of breast cancer. Conversely, the work presented here centers primarily on multi-view feature fusion based on EfficientNet, which proves to be computationally efficient while offering strong performance and good interpretability for the clinically complex task of BI-RADS B vs. C classification.

### 5.4. Statistical Robustness

The multi-view concatenation model exhibited slightly better performance than the single-view models, with an overall comparable discriminatory behavior across configurations. In addition, the patient-based evaluation revealed consistent predictions aligned with ground-truth annotations, implying a stable model behavior across all test cases.

Furthermore, the ablation study revealed relatively small differences between the configurations, suggesting that both multi-view fusion and U-Net refinement can contribute to performance enhancements, without one component clearly dominating.

McNemar’s test for classification performance and DeLong’s test for the comparison of AUCs were employed for the purpose of evaluating the statistical robustness in more detail. The proposed model exhibited statistically substantial enhancements over the evaluated baselines, particularly the multi-average fusion strategy (McNemar: *p* = 0.0024; DeLong: *p* = 0.0352).

The robustness of the results was further corroborated by the bootstrap analysis (2000 resamples), with systematically small confidence intervals through all metrics.

The single-view models present average discrimination capacities. This indicates that CC and MLO views offer complementary density information. Furthermore, the single-view reference methods were systematically surpassed by feature-level fusion, which further supports the benefit of employing joint multi-view representation learning.

Multi-means fusion improves AUC and sensitivity, which justifies the interest in combining views, even in cases where spatial interactions between features are not clearly preserved. Conversely, feature-level concatenation offers more practical inter-view integration, which contributes to enhanced discrimination performance.

However, the ablation results presented in Table 9 indicate that performance differences between the proposed model and intimately linked fusion strategies are insignificant, with overlapping confidence intervals, which implies that the interpretation of these enhancements must be made with caution.

Overall, the outcomes highlight the stable and robust performance of the proposed pipeline within the assessed protocol, with statistically supported improvements compared to reference methods.

### 5.5. Considerations Regarding Interpretability

Grad-CAM visualizations confirmed that the model focuses essentially on fibroglandular parenchymal regions in preference to irrelevant background structures.

The assessment interpretability was carried out by two radiologists. One of them was an independent expert who had not participated in the development of the model. Although this method diminishes subjectivity compared to a single-reader evaluation, the small number of cases evaluated represents a constraint. This vindicates conducting future validation studies with multiple readers.

### 5.6. Calibration and Clinical Deployment

The probability calibration analysis showed good agreement between the predicted probabilities and observed results, suggesting potential applicability to clinical decision support systems. Nevertheless, prospective validation in real-world clinical practice is necessary before actual deployment. It is also important to emphasize that this model is designed as a support tool; it is not a substitute for radiologists’ expertise.

### 5.7. Limitations

Dataset reduction and potential selection bias: Exclusion of implant cases and incomplete records reduced the dataset, which could have limited anatomical and acquisition variability.

Task formulation: This study focuses on binary BI-RADS B vs. C classification, restraining its applicability across the entire density spectrum.

External validation: Independent multicenter testing is strongly recommended to guarantee robustness among heterogeneous populations.

Interpretability assessment: Grad-CAM evaluation was performed based on a randomly selected, class-stratified subset of 100 test cases. It was then evaluated by two board-certified radiologists, one of whom was an independent expert. Therefore, larger, multi-reader validation studies involving completely independent radiologists are highly recommended to improve the generalizability of the results.

Statistical significance: Although the proposed framework achieved the highest accuracy and area under the curve (AUC) among the evaluated configurations, formal statistical testing showed substantial differences over the assessed baselines (as indicated in Section 5.4), even though differences between intimately linked ablation configurations were very small. Therefore, the observed performance gains should be interpreted with caution, and they also need to be validated in more detail on external datasets.

### 5.8. Future Directions

Future developments could involve multi-task learning combining density assessment with lesion detection, prospective clinical validation studies, and evaluation on external datasets. These extensions may enhance robustness, interpretability, and translational relevance even more. Moreover, the quantification of uncertainty and external multicenter validation could enhance the clinical generalizability and reliability of the model. Overall, this work highlights the significance of AI-based decision support systems in ambiguous clinical settings characterized by high inter-observer variability.

## 6. Conclusions

An automated, interpretable, and integrable pipeline within a CAD system is presented in this work for the binary classification of B and C breast densities. It is based on the EfficientNet–UNet hybrid architecture.

The main contributions are given below:

1. Multi-scale representation: The simultaneous extraction of fine and global breast parenchymal features optimizes the discrimination of intermediate densities. This approach is compatible with image preprocessing within a CAD pipeline.
2. (CC+MLO) Multi-view fusion: Improved accuracy and intra-patient consistency support full exploitation of the complementary information from each view and facilitate integration into the clinical workflow.
3. Clinical interpretability: The Grad-CAM technique is used to visualize all contributive areas, ensuring transparent decision-making and supporting radiologists’ qualitative assessment in clinical decision support settings.

Competitive performance: The accuracy (87%) and AUC (94.40%) under the evaluated protocol reveal that competitive performance is achieved by the model. Furthermore, significant improvements compared to the evaluated reference values were confirmed with statistical analysis. However, the differences between highly comparable ablation configurations are limited. Therefore, performance differences should be interpreted cautiously.

4. Probability calibration: The predicted probabilities were calibrated, guaranteeing more reliable risk estimation suitable for clinical decision support.

These findings explicitly demonstrate that multi-view, multi-scale image preprocessing, coupled with interpretability and integration into a CAD system, provides a promising clinically validated framework for automated breast density assessment.

This study successfully demonstrates the potential of artificial intelligence to help radiologists reduce variability and improve the reliability of breast density assessment in real-world screening settings. This was achieved through a combination of multi-view analysis of mammograms with interpretable deep learning models and comprehensive and rigorous medical assessment. The method adopted herein paves the way for promising future developments, including multimodality fusion, optimization for real-time inference, and deployment in clinical workflows. This approach effectively addresses the current challenges of quantitative medical imaging and breast cancer screening, especially with regard to population-level screening programs.

Although implant cases were excluded to ensure methodological consistency, future large-scale validation including heterogeneous acquisition conditions will be essential for confirming the robustness and generalizability of the proposed framework.

## Figures and Tables

**Table 1:** Image distribution and class proportions in each set.

Set	Images	Distribution (0/1)
Training	18,877	51.96%/48.04%
Validation	2315	51.14%/48.86%
Test	2321	51.79%/48.21%

**Table 2:** Model performance on the test set.

Model	Accuracy (%)	AUC (%)	F1-Score (%)
EfficientNet-UNet hybrid model (proposed model)	87%	94.40%	85.80%
ResNet-50	84.33%	91.80%	84.80%
Vision Transformer	84.80%	92.00%	85%

**Table 3:** Per-class performance metrics across the test set.

Class	Precision (%)	Recall (%)	F1-Score (%)
B (0)	86.98%	89.02%	87.99%
C (1)	86.62%	85%	85.80%

**Table 4:** Analysis of false positive (FP) cases.

Set	Type	GT	Pred	Radiological Feature	Grad-CAM Focus	Interpretation	Agreement
FP1	FP	B	C	Focal fibroglandular density	Localized fibroglandular activation	Overestimation due to focal density emphasis	Full
FP2	FP	B	C	Partial glandular distribution	Fragmented asymmetric activation	Misinterpretation of heterogeneous tissue	Full
FP3	FP	B	C	Mild tissue overlap	Activation in overlapping anatomical regions	Spatial ambiguity leads to false density perception	Full
FP4	FP	B	C	Peripheral density focus	Strong peripheral activation	Peripheral bias affecting global assessment	Full
FP5	FP	B	C	Moderate focal opacity	Localized high-intensity response	Saliency-driven false positive activation	Full

**Table 5:** Analysis of false negative (FN) cases.

Set	Type	GT	Pred	Radiological Feature	Grad-CAM Focus	Interpretation	Agreement
FN1	FN	C	B	Subtle diffuse density	Weak scattered activation	Low-contrast pattern not detected	Full
FN2	FN	C	B	Heterogeneous glandular pattern	Diffuse inconsistent activation	Structural complexity reduces sensitivity	Full
FN3	FN	C	B	Subtle focal density	Weak localized activation	Small lesion under-detected	Partial
FN4	FN	C	B	High breast density	Low-contrast diffuse activation	Dense tissue reduces discriminability	Full
FN5	FN	C	B	Poor glandular definition	Non-specific activation	Lack of structured features	Full

**Table 6:** Distribution of expert evaluation scores for Grad-CAM alignment (*N* = 100).

Alignment Category	Score	Radiologist 1 (%)	Radiologist 2 (%)	Consensus
Fully aligned	2	52	50	54
Partially aligned	1	33	33	31
Misaligned	0	15	17	15
Clinically acceptable alignment (Full + Partial)		85	83	85

**Table 7:** Comparison of agreement and performance metrics.

System/Evaluation	Metric	Value
Radiologist inter-reader agreement	Cohen’s κ	0.82
Model vs. consensus (explainability)	Weighted κ	0.85
Model classification performance	AUC	0.944
Model vs. consensus agreement	Accuracy	0.87%

**Table 8:** Comparison with state-of-the-art methods regarding the breast density classification. The above results are provided for contextual comparison only because disparities in datasets, class definitions, and evaluation protocols (image-wise vs. patient-wise) restrict the rigorous numerical comparability.

Method	Dataset	Dataset Type	Task	Evaluation Protocol	Multi-View Strategy	Accuracy (%)	AUC	Interpret- Ability	Notes
TwoViewDensityNet [27]	DDSM	Curated (small-scale)	4-class BI-RADS	Image-wise	CC+MLO dual-view	95.83	99.51	NR	Public benchmark study
TwoViewDensityNet [27]	INbreast	Curated (small-scale)	4-class BI-RADS	Image-wise	CC+MLO dual-view	96.00	97.44	NR	Controlled experimental setting
Local septenary pattern [42]	MIAS/INbreast	Curated	Binary density	Image-wise	Single view	MIAS: 83.3, INbreast: 80.5	NR	NR	Handcrafted feature-based approach
ResNet-50 (Ours)	RSNA	Real-world, large-scale	Binary BI-RADS B vs. C	Patient-wise	Single view	84.30	87.20	NR	Trained under patient-wise protocol
Vision Transformer (Ours)	RSNA	Real-world, large-scale	Binary BI-RADS B vs. C	Patient-wise	Single view	83.20	88.00	NR	Trained under patient-wise protocol
EfficientNet–Net Hybrid (Ours)	RSNA (4796 patients, 23,513 images)	Real-world, large-scale	Binary BI-RADS B vs. C	Patient-wise	Feature level CC+MLO fusion	87.00	94.39	Grad-CAM	Multi-view, Interpretable, patient-wise

**Table 9:** Results of the ablation study: accuracy, AUC, and sensitivity for different model configurations.

Configuration	Accuracy (95% CI)	AUC (95% CI)	F1-Score (95% CI)	Sensitivity (95% CI)	Interpretation
CC-only	0.856 (0.838–0.874)	0.939 (0.921–0.957)	0.853 (0.835–0.871)	0.858 (0.840–0.876)	Single-view baseline
MLO-only	0.855 (0.837–0.873)	0.934 (0.916–0.952)	0.846 (0.828–0.864)	0.856 (0.838–0.874)	Single-view baseline
Multi-view (EfficientNet concat, no U-Net)	0.865 (0.847–0.883)	0.931 (0.923–0.959)	0.850 (0.832–0.868)	0.856 (0.840–0.876)	Feature-level fusion without segmentation refinement
EfficientNet + U-Net (CC only, no multi-view fusion)	0.862 (0.842–0.878)	0.937 (0.922–0.958)	0.853 (0.835–0.871)	0.848 (0.830–0.866)	Single-view segmentation- enhanced model
Multi-view average	0.868 (0.850–0.886)	0.942 (0.924–0.960)	0.863 (0.845–0.881)	0.893 (0.875–0.911)	Improved sensitivity
Backbone-only	0.864 (0.846–0.882)	0.940 (0.922–0.958)	0.852 (0.834–0.870)	0.823 (0.805–0.841)	Good global base
Proposed (Full: U-Net + CC+MLO concat)	0.870 (0.852–0.888)	0.944 (0.926–0.962)	0.858 (0.840–0.876)	0.864 (0.846–0.882)	Multi-view model indicating the highest Accuracy and AUC among the evaluated configurations.

## Data Availability

The data used in this study are publicly available in the RSNA Breast Cancer Screening Mammography Dataset, accessible via the Kaggle platform.
